# Modeling and Optimization of Hydraulic and Thermal Performance of a Tesla Valve Using a Numerical Method and Artificial Neural Network

**DOI:** 10.3390/e25070967

**Published:** 2023-06-22

**Authors:** Kourosh Vaferi, Mohammad Vajdi, Amir Shadian, Hamed Ahadnejad, Farhad Sadegh Moghanlou, Hossein Nami, Haleh Jafarzadeh

**Affiliations:** 1 Department of Mechanical Engineering, University of Mohaghegh Ardabili, Ardabil 5619913131, Iran; k.vaferi@uma.ac.ir (K.V.); hamed.ahadnejad@outlook.com (H.A.); 2 Department of Mechanical Engineering, University of Tabriz, Tabriz 5166616471, Iran; amir.shadian99@ms.tabrizu.ac.ir; 3 SDU Life Cycle Engineering, Department of Green Technology, University of Southern Denmark, Campusvej 55, 5230 Odense M, Denmark; 4 Department of Civil Engineering, School of Science and Engineering, Khazar University, Baku 1096, Azerbaijan; haleh_jafarzadeh@yahoo.com

**Keywords:** Tesla valve, optimization, diodicity, thermo-hydraulic performance, artificial neural network

## Abstract

The Tesla valve is a non-moving check valve used in various industries to control fluid flow. It is a passive flow control device that does not require external power to operate. Due to its unique geometry, it causes more pressure drop in the reverse direction than in the forward direction. This device’s optimal performance in heat transfer applications has led to the use of Tesla valve designs in heat sinks and heat exchangers. This study investigated a Tesla valve with unconventional geometry through numerical analysis. Two geometrical parameters and inlet velocity were selected as input variables. Also, the pressure drop ratio (PDR) and temperature difference ratio (TDR) parameters were chosen as the investigated responses. By leveraging numerical data, artificial neural networks were trained to construct precise prediction models for responses. The optimal designs of the Tesla valve for different conditions were then reported using the genetic algorithm method and prediction models. The results indicated that the coefficient of determination for both prediction models was above 0.99, demonstrating high accuracy. The most optimal PDR value was 4.581, indicating that the pressure drop in the reverse flow direction is 358.1% higher than in the forward flow direction. The best TDR response value was found to be 1.862.

## 1. Introduction

Non-moving-part valves (NMPVs) are efficient equipment used as passive fluid controllers. Compared to conventional check valves with moving parts, NMPVs have advantages in terms of manufacturing and do not require external power to operate [1]. One specific type of NMPV is the Tesla valve. The Tesla valve, first introduced by Nikola Tesla in 1920, is a check valve with no moving parts in its structure and is also known as a fluid diode [2,3,4]. This valve allows fluid to flow easily in the forward direction but prevents fluid from flowing in the reverse direction. Due to its unique construction, the Tesla valve causes a lower pressure drop in the forward direction than in the reverse direction [5]. In fact, Tesla valves can be considered one-way valves. Due to the demand for passive fluid flow control, especially in mini and micro scales, the utilization of Tesla valves is becoming increasingly attractive to researchers. These valves are widely utilized in industries for controlling flow rate and direction in equipment such as internal combustion engines [6], turbines [7], pumps [8], and compressors [9,10]. Tesla valves can also be used in mini and microfluid applications, such as micromixers [11], and for the decompression process in hydrogen fuel cells [12,13]. Furthermore, their thermal characteristics make them suitable for heat transfer applications, especially in heat sinks for battery cooling [14,15].

De Vries et al. [16] designed a new construction for the Tesla valve to improve fluid flow and reduce thermal resistance in a pulsating heat pipe (PHP). They experimentally investigated its diodicity and operation by steady two-phase flow and laminar single-phase modeling. Laminar single-phase modeling demonstrated that the new construction of the Tesla valve generates more diodicity than other conventional Tesla valves at low Reynolds numbers. In addition, they found a 14% decrease in thermal resistance for the PHP with Tesla valves compared to similar PHP without Tesla valves in their structure. Jin et al. [12] numerically investigated the hydrogen decompression process using a Tesla valve with reverse flow direction for a wide range of inlet velocities. The results of their study highlighted that a large valve angle, small inner curve radius, and small hydraulic diameter could offer a high Δ*P*. Qian et al. [17] investigated the exergy loss and the possibility of aerodynamic noise occurrence in a Tesla valve with hydrogen fluid flow used for decompression. To perform these analyses, they applied changes in the valve inlet and outlet pressure ratio and the number of valve stages. They reported that increasing the pressure ratio raises the Ma and exergy loss. Also, they found that the Ma increases and exergy loss decreases by increasing the stage number of the valve. Monika et al. [15] presented a novel configuration for a multi-stage Tesla valve. They numerically analyzed it to investigate the temperature gradient created in a cold plate with a Tesla channel for the thermal management of Li-ion batteries. They observed that their new design provides more efficient cooling than conventional channels by improving the heat transfer rate of the cold plate. Liu et al. [2] presented a symmetrical design for the bent channel structure of the Tesla valve and investigated the hydraulic characteristics of the fluid using the finite element method (FEM). The results indicated that by enhancing the symmetry of the structure, the hydraulic diodicity performance of the valve increases. Bao and Wang [10] improved the relative pressure drop ratio (RPDR) and absolute pressure drop ratio (APDR) parameters to compensate diodicity performance of the Tesla valve. They designed a novel Tesla valve with special tapering and widening in its body and compared it with other types of Tesla valves. Their results stated that the novel presented design has a better APDR than conventional Tesla valves, and it was also found that this parameter increases linearly with the increase in the number of valve stages while the RPDR gradually reaches a constant value. In this study, they also investigated the thermal diodicity and observed that this parameter increases with increasing velocity and number of stages, but it is independent of wall and inlet temperatures. Lu et al. [14] presented a cold plate cooling system inspired by a Tesla valve for the enhancement of cooling in batteries. The optimization results showed that under specific geometrical conditions and a velocity of 0.83 m/s, the cold plate with Tesla valve channels and reverse flow establishes a good equilibrium between thermal performance and energy consumption. Yang et al. [18], using computational fluid dynamics (CFD) and conducting experiments, designed a new micromixer with a Tesla valve structure to obtain an effective mixing process in microfluidic equipment for biological applications. They observed that the mixing performance is more suitable for Reynolds numbers ranging from 0.1 to 100. Sun et al. [19] numerically analyzed a microchannel heat sink with Tesla valve-shaped channels. By examining the thermo-hydraulic performance, they realized that using the channels with Tesla valve design instead of the smooth channel increases the Nusselt number by 102.3%, and the friction factor increases by 3.21 times.

Artificial neural network (ANN) is a technology inspired by the brain and biological nervous system that mimics their electrical activity. This method forms the core of deep learning algorithms and is a subset of machine learning. One of the main benefits of ANN over other models is its ability to represent a multivariable problem based on the complex interactions between the variables and extract implicit nonlinear correlations among them [20,21,22]. This model-optimization method is widely used across various fields due to its impressive performance [23,24,25,26,27].

Polat and Cadirci [28] investigated the heat transfer of a microchannel heat sink with a diamond-shaped pin fin array under laminar, steady-state, and incompressible flow boundary conditions. They utilized a multi-layer ANN model coded in Python and trained with CFD outcomes to investigate Nusselt and Poiseuille numbers representing thermal and hydrodynamic features. The results showed that the pin-fin angle has the greatest impact on Nusselt and Poiseuille numbers. Kanesan et al. [29] developed a response model for thermal energy storage heat sinks, which are commonly used to cool electronics. They examined an aluminum heat sink for thermal energy storage (TES) using paraffin as the phase change material (PCM). By combining the trained ANN model with the genetic algorithm (GA) method, they optimized the variables related to the TES heat sink’s geometry and the used PCM volume. This research demonstrates that combining ANN with GA creates a more effective optimization tool. Mahmoudabadbozchelou et al. [30] studied the enhancement of the heat transfer rate of impinging jets by adding nanoparticles to the background fluid. They utilized ANN and GA methods to optimize the uniform cooling of a continuously heated surface. The results indicated that the addition of nanoparticles to water led to an increase in heat transfer due to the increased thermal conductivity of the fluid, and larger particle sizes and concentrations caused a further increase. Kuang et al. [31] investigated the heat transfer in the boiling process of hydrogen flow and used ANN to identify the most influential parameter in this process. The study found that the boiling number is one of the most critical factors in determining the boiling heat transfer coefficient. The researchers also observed that the effect of saturation pressure on the flow boiling heat transfer coefficient is more significant than its effect on the flow rate of liquid hydrogen. Yunn Heng et al. [32] proposed a rapid and reliable transient thermal prediction technique for estimating the exit temperature of a parabolic trough collector tube. ANN was applied to analyze the increase in exit temperature produced by a single heat flux pulse. They observed that the outcomes could be utilized for preliminary system planning, heat balance assessment, and systems engineering. The study reported that this method works well with changing and steady solar radiation, making it useful for designing parabolic trough technologies in any weather condition worldwide. Ermis et al. [33] investigated phase change heat transfer in a finned-tube latent heat thermal storage system using an ANN approach. The trained ANN model predicted the total quantity of stored heat with an average error of 5.58%, resulting in a more accurate heat storage estimation than the numerical model results. In another study, Xie et al. [34] evaluated multi-layer neural network designs based on experimental datasets of Nusselt number and friction factor for three heat exchangers. Their findings demonstrated that the ANN method performs well in predicting heat transfer and fluid flow for laminar or turbulent regimes in such heat exchangers, such that the variance between their study’s predicted and experimental results was approximately 4%. Their work suggests that ANNs can be utilized for thermal system performance anticipation, particularly heat exchanger modeling for heat transfer assessment. Beigmoradi et al. [35] conducted a study on the aerodynamic optimization of the rear end of a car using ANN. They selected several geometric parameters as input variables and studied the drag coefficient and maximum acoustic power level as responses. The Taguchi method was used to reduce the number of tests, and the GA method was used to optimize the model. The results indicate that the drag coefficient decreases with the increase in the rear box length parameter, but it leads to an increase in the acoustic power response. Li et al. [36] used three ANNs to predict the properties of China RP-3 kerosene at a faster rate. Their results show that the properties predicted by ANN models have high accuracy and are consistent with the calculations of the extended corresponding state principle method. They also observed that the prediction of properties is 10^4^ times faster than the calculations, which is a significant achievement. George et al. [37] optimized the design of a multi-layer porous wave absorber by using the ANN method and a data set consisting of 200 combinations. The trained prediction model in their work has a determination coefficient of 0.97, indicating high accuracy in predicting results. They found that the optimal range of design variables for submergence depth was 0.055–0.067, the distance between plates was 0.064–0.080, and porosity was 0.117–0.173. Zhu et al. [38] investigated the total output power due to changes in the configuration of the array of wave energy converters. Using the ANN method, they trained a prediction model for the desired response. They reported that using energy converters with shorter distances improves energy absorption and was also suitable and beneficial for engineering applications.

The present study investigated a two-stage Tesla valve with an unconventional geometry numerically. The parameters of the divider baffle length (*L*), step length (*S*), and inlet velocity of the valve (*V*) were considered as input variables. Also, the diodicity (PDR) and the ratio of the temperature difference (TDR) in reverse and forward directions were selected as the responses. Of course, due to the consistency of the properties and mass flow rate of the passing fluid in both directions, TDR also indicates the heat transfer ratio. Numerous numerical experiments were conducted under different conditions to obtain the prediction models for each response, and the results were used to train artificial neural networks. These models can predict the values of the responses accurately and quickly without the need for complex calculations or additional experiments. Finally, the optimal conditions and designs of the Tesla valve for various applications were determined using the genetic algorithm method and the obtained prediction models. This approach can significantly reduce the time and cost required for designing and optimizing the Tesla valve for specific applications.

## 2. Methodology

### 2.1. Tesla Valve Structure

**Figure 1:**
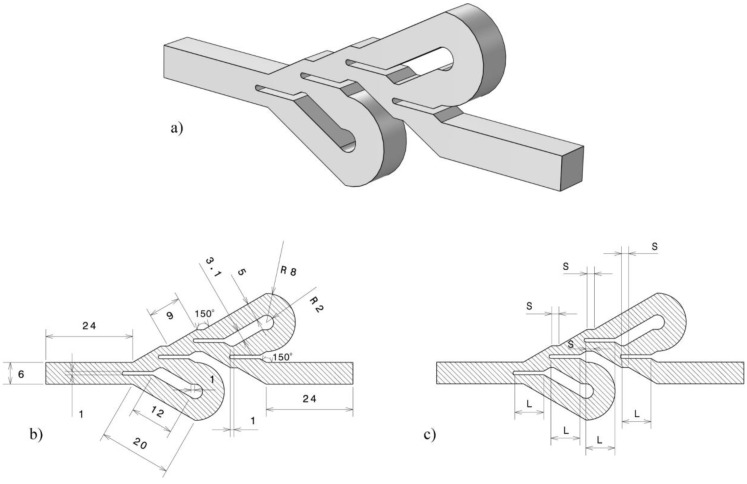
(**a**) 3D view of the investigated geometry, (**b**) fixed geometric parameters, (**c**) variable geometric parameters.

According to the results of the research conducted on Tesla valves and preliminary analysis, a specific structure for this device was proposed, which was based on the design by Bao and Wang [10]. The physical shape and geometrical characteristics of the Tesla valve structure are shown in Figure 1. A three-dimensional view of the intended structure is depicted in Figure 1a. The constant and variable dimensions of the simulated geometry are shown in Figure 1b and Figure 1c, respectively. The most significant difference between this Tesla valve and other conventional Tesla valves is the use of a unique three-way pattern that consists of a divider baffle. In this work, the divider baffle length (*L*) and step length (*S*) were considered as the two geometrical variables to investigate the performance of the Tesla valve. It should also be noted that in addition to the variables mentioned, the effects of the velocity of the input flow to the Tesla valve were investigated.

**Figure 2:**
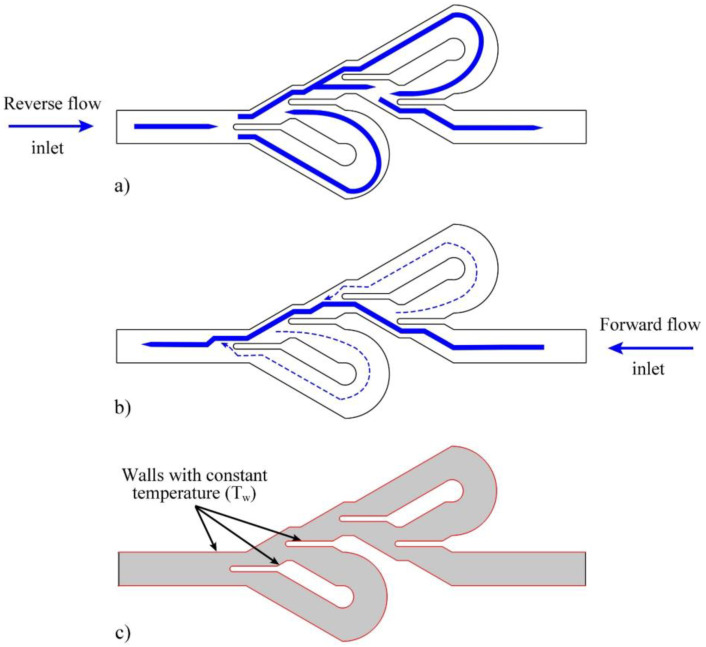
(**a**) The reverse fluid movement pattern in the Tesla valve, (**b**) the forward fluid movement pattern in the Tesla valve, (**c**) the thermal boundary condition in the Tesla valve.

The fluid movement pattern in this unconventional design for the Tesla valve in reverse and forward directions is based on Figure 2a,b, respectively. When the fluid enters the valve in the reverse direction, the dividers direct part of the fluid flow into the bent channels. While with the movement of the fluid in the forward direction, the main flow of the fluid can easily pass through the main channel of the valve. Therefore, it is expected that due to more friction and fluid interaction in the reverse direction, a greater Δ*P* will occur in this direction.

### 2.2. Assumptions and Boundary Conditions

All numerical analysis and simulation processes were performed using FEM in COMSOL Multiphysics software. Since this study aims to enhance the diodicity and TDR of the Tesla valve, water fluid was simulated under special conditions. Consequently, several boundary conditions and assumptions were applied to achieve an appropriate result. Boundary conditions and assumptions are as follows:

- This study was investigated in a steady state;
- The fluid passing through the Tesla valve was turbulent, single-phase, and incompressible;
- The thermophysical characteristics of the water were considered to be constant, as listed in Table 1;
- No-slip and no-temperature-jump conditions were assumed for the walls in contact with the fluid;
- The wall’s temperature around the fluid was adopted constant and equal to 350 K (Figure 2c);
- The water inlet temperature was considered constant and equal to 293.15 K;
- The gauge pressure of the Tesla valve outlet was considered zero.

### 2.3. Governing Equations

The present study numerically investigated the fluid flow and heat transfer inside the designed Tesla valve using CFD in two dimensions. Governing equations that must be solved for determining thermo-hydraulic parameters of the flow are conservation equations and *k-ε* turbulence model equations. For an incompressible viscous flow, the first equation is the mass conservation equation, and it can be expressed as follows [10,39]:

(1) ρ∇·V=0

The momentum conservation equation is written as follows:

(2) $$\rho V\cdot \nabla V=-\nabla P+\nabla \cdot \left(\left(\mu +\mu_{t}\right)\left(\nabla V+{\left(\nabla V\right)}^{T}\right)\right)$$

Also, the energy conservation equation is given by:

(3) $$\rho C_{p}V\cdot \nabla T=\nabla \cdot \left(\left(\lambda +\frac{\mu_{t}}{Pr}\right)\nabla T\right)$$

where *V*, *μ*, *μ_t,_* and *λ* represent the velocity, dynamic viscosity, turbulent viscosity, and thermal conductivity, respectively. In this research, the standard *k-ε* turbulent model was employed in order to analyze the flow in the valve. This turbulence model is the most prevalent model used in CFD to represent the mean flow characteristics of turbulent flow. It is a two-equation model that uses two transport equations to provide a general description of turbulence. The turbulent kinetic energy and specific dissipation rate for turbulent flow in the standard *k-ε* model are defined by Equations (4) and (5), respectively [40,41,42].

(4) $$\rho V\cdot \nabla k=\nabla \cdot \left(\left(\mu +\frac{\mu_{t}}{\sigma_{k}}\right)\nabla k\right)+P_{k}-\rho \varepsilon$$

(5) $$\rho V\cdot \nabla \varepsilon =\nabla \left(\left(\mu +\frac{\mu_{t}}{\sigma_{\varepsilon }}\right)\nabla \varepsilon \right)+C_{\varepsilon 1}\frac{\varepsilon }{k}P_{k}-C_{\varepsilon 2}\rho \frac{\varepsilon^{2}}{k}$$

(6) $$\mu_{t}=\rho C_{\mu }\frac{k^{2}}{\varepsilon }$$

(7) $$P_{k}=\mu_{t}\left(\nabla V:\left(\nabla V+{\left(\nabla V\right)}^{T}\right)\right)$$

where *k* and *P_k_* represent the turbulent kinetic energy and the production of this energy due to the mean velocity gradients, respectively, and the parameter *ε* is the energy dissipation rate obtained in the turbulent flow. Also, the constants related to the turbulence model are presented in Table 2.

In the present study, the segregated approach, a pressure-based solver, was used to solve the governing equations. As implemented in COMSOL Multiphysics, this approach solves the velocity and pressure in one step. In contrast, other variables, such as temperature, are solved separately in other steps. This solver configuration allows for the decoupling and independent solution of different variables, which can help improve computational efficiency and convergence [43]. This research used a parallel direct sparse solver (PARDISO) in numerical simulations. The PARDISO solver is a state-of-the-art direct sparse solver in computational science and engineering. This solver is known for its efficiency, scalability, and ability to handle large-scale linear systems from various numerical simulations. It employs advanced algorithms and parallel computing techniques to efficiently handle the matrix factorization and solve the system of equations [44,45,46].

Diodicity is an essential factor that evaluates the Tesla valve’s hydraulic performance. This parameter highlights the effectiveness of the valve based on the ratio of Δ*P* in reverse flow to forward flow in an identical flow rate [1]. By increasing diodicity, the performance of this device as a check valve will be improved. On the other hand, if this device is used in thermal applications, the thermal parameters of this device should be improved. The hydraulic and thermal diodicity of the Tesla valve were presented as PDR (pressure drop ratio) and TDR (thermal difference ratio), respectively, which were calculated as follows:

(8) $$PDR=\frac{\Delta P_{r}}{\Delta P_{f}}=\frac{{\left(P_{in}-P_{out}\right)}_{r}}{{\left(P_{in}-P_{out}\right)}_{f}}$$

(9) $$TDR=\frac{\Delta T_{r}}{\Delta T_{f}}=\frac{{\left(T_{out}-T_{in}\right)}_{r}}{{\left(T_{out}-T_{in}\right)}_{f}}$$

### 2.4. Mesh Independency

**Figure 3:**
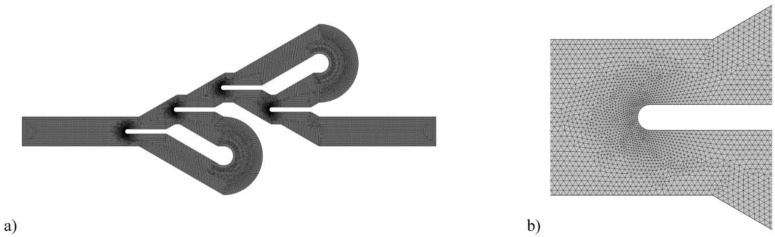
(**a**) Overview and (**b**) refined mesh near the divider baffle for the computational domain.

COMSOL Multiphysics was used to develop a two-dimensional triangular mesh type, as seen in Figure 3. In this simulation, the average mesh quality is 0.93. According to the statistics in Table 3, the thermal and hydraulic results of the numerical simulation with the number of 50.44 elements per 1 mm^2^ are independent of the mesh. As can be seen, by increasing the mesh elements number to 88.58 per 1 mm^2^, the time to solve the simulation increases by 56%, while the results related to the temperature difference and pressure drop change by 1.23% and 0.44%, respectively.

### 2.5. Simulation Method Validation

**Figure 4:**
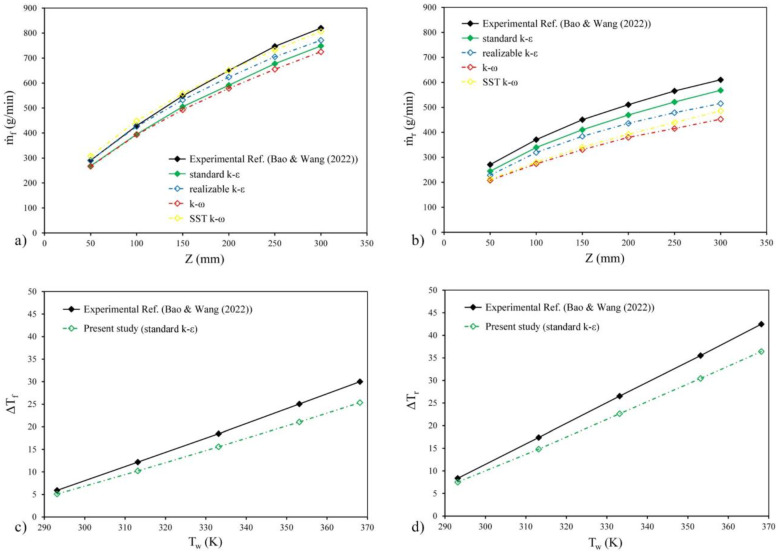
Comparison of results calculated by the numerical method of the present work with the experimental study of Bao and Wang [10]: (**a**) the hydraulic results in the forward flow, (**b**) the hydraulic results in the reverse flow, (**c**) the thermal results in the forward flow, and (**d**) the thermal results in the reverse flow.

Since this work is a numerical study, it needs to be validated by a reliable experimental reference. Therefore, all processes and numerical simulations in the present study were based on the experimental research conducted by Bao and Wang [10], and the results of this work were compared with their research findings to ensure the accuracy and validity of the analysis done in this work. In the present work, the thermo-hydraulic performance of the equivalent shunts (ES) Tesla valve with six stages was investigated and compared with the reference study [10]. Figure 4 shows that both hydraulic and thermal results obtained from the numerical analysis have a reasonable correlation with the findings provided in the experimental reference study, indicating that the numerical method adopted in this research has reasonable accuracy. It is worth noting that in the subsequent part of this work, a two-stage Tesla valve was designed instead of the six-stage valve to allow for physical changes and further investigations.

### 2.6. Turbulent Model Validation

In this research, different turbulence models were tested and compared with the experimental results to select the turbulence model used in the numerical analysis, according to Figure 4a,b. Equation (10) was used to calculate the average error of numerical analysis for each turbulence model. According to the results of the numerical analysis using the *k-ω* turbulence model, the average error of the hydraulic results in the reverse flow was 25.7%, and it was 10.2% in the forward flow. However, when the *SST k-ω* model was used in the simulations, the average errors for the reverse and forward directions results were reduced to 22.7% and 2.6%, respectively. The realizable *k-ε* model showed an average error of 15% in the reverse flow and 3.3% in the forward flow for the results obtained in the analysis of the Tesla valve. On the other hand, the standard k-ε model gave an average error of 8.3% and 8.4% for hydraulic results in the reverse and forward directions, respectively. Based on these results, it can be concluded that the standard *k-ε* model is the most appropriate turbulence model to use in numerical simulations because it gives suitable and close each other average errors in both directions.

The *y+* is a dimensionless parameter similar to the local Reynolds number used in CFD to characterize the near-wall flow behavior by quantifying the distance from the solid wall to the nearest mesh element’s center. This parameter is used as a criterion to evaluate the appropriateness of the grid element size on the walls’ borders. On the other hand, it plays a crucial role in selecting an appropriate wall modeling approach, such as wall functions or low Reynolds number (LRN) models, based on the flow regime and the desired level of accuracy for capturing the near-wall physics. This parameter is calculated according to Equations (11) and (12). In these equations, *V_τ_* and *τ_w_* represent the friction velocity and wall shear stress, respectively. Also, *y* is the distance between the wall and the center of the nearest mesh element to the wall. Higher *y+* values (often above 30) indicate fully turbulent flows where wall functions are effective. In comparison, lower *y+* values suggest laminar or transitional flows requiring more refined modeling techniques to resolve the near-wall region accurately [47]. The calculation of *y+* was performed across different turbulence models, and the results are presented in Table 4. It is evident from the data that the *y+* values almost in all cases exceed 30, indicating a predominantly turbulent near-wall flow regime.

According to the *y+* parameter results, it can be seen that the use of the wall functions model was suitable for investigating the fluid flow behavior near the walls in the present study. These wall functions are derived from empirical correlations based on experimental data, and *k-ε* models widely use these wall functions to capture the turbulence characteristics near walls. Also, because the values of *y+* were above 30, using the LRN wall model, which is used in the *k-ω* and *SST k-ω* turbulence models, was unsuitable for use in the present work [48,49]. Further, considering that the numerical results of the standard *k-ε* turbulence model were in better agreement with the experimental results compared to the realizable *k-ε* model, the standard *k-ε* model was used for further investigations in this work.

(10) $$\bar{Error}=100\times \frac{1}{m}\sum_{i=1}^{m}\frac{\left|r_{num}-r_{exp}\right|}{r_{exp}}$$

(11) $$y^{+}=\frac{\rho V_{\tau }y}{\mu }$$

(12) $$V_{\tau }=\sqrt{\frac{\tau_{w}}{\rho }}$$

### 2.7. Artificial Neural Network Approach

One way to reduce computational time and save financial resources is by using black-box methods, which deal with input and output data without considering the possible physical processes. A practical approach in this field is the artificial neural network (ANN), inspired by the human nervous system. Neurons are the fundamental processors in neural networks, and each neuron may receive multiple inputs from other neurons and have one or more outputs based on its activity [33].

**Figure 5:**
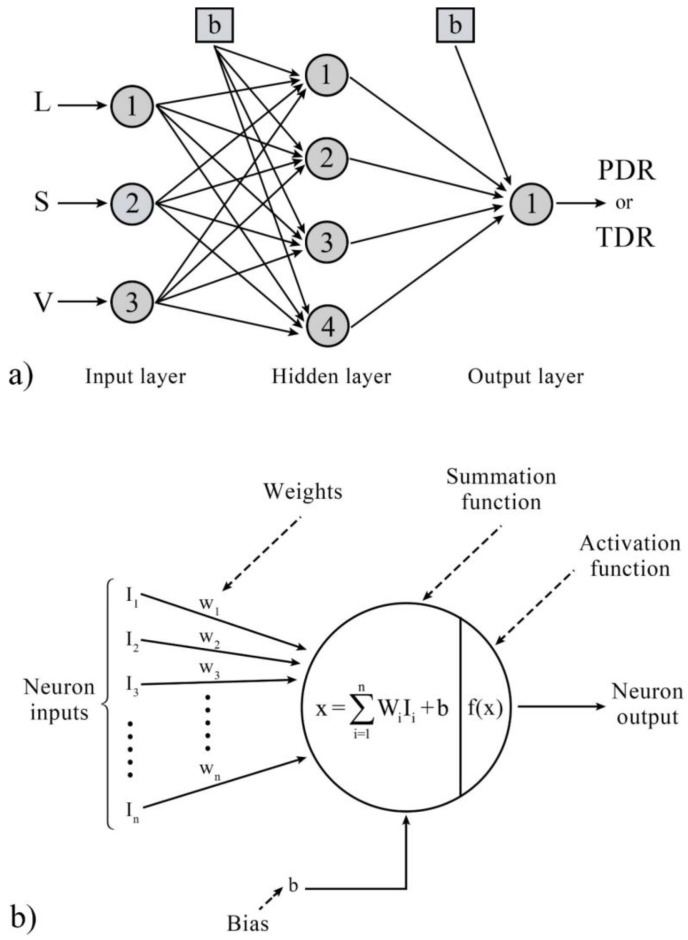
(**a**) The three-layer structure of the ANN network was used in the present work, (**b**) calculations performed in neurons of the hidden and output layers.

In this study, a separate three-layer structure was designed for each response (TDR and PDR), consisting of input, hidden, and output layers, as shown in Figure 5a. The input layer has three neurons that serve as the network’s inputs. The hidden layer has four neurons, and the output layer contains one neuron. Based on the investigations conducted, it was observed that prediction models with fewer than four neurons in the hidden layer did not have satisfactory performance. Conversely, an excessive increase in neurons in this layer led to overfitting in the model’s results. The activation functions must be derivable to perform the backpropagation function in model training. Therefore, the tangent-sigmoid activation function was used in hidden layer neurons, and the linear activation function was used in the output layer neuron to obtain the response value. To better understand the performance of each neuron in the hidden and output layers, Figure 5b was presented, which shows the performed calculations.

MATLAB (2016b) software was applied to optimize models using the ANN approach, and the Levenberg–Marquardt method was employed to train artificial neural networks by utilizing the available data. The variables and their ranges for simulation and optimization were tabulated in Table 5, and these ranges were selected based on the geometric and boundary conditions. Furthermore, Table 6 presents a list of numerical tests selected using the central composite design (CCD) method, which can minimize the number of tests, which is an important aspect, especially in experimental studies [50]. To ensure the development of reliable prediction models, a careful data allocation strategy was employed. As illustrated in Table 6, 70% of the data was allocated for training the models, 15% for validation purposes, and 15% for testing the models. Notably, this classification was performed randomly, ensuring an unbiased data distribution. To assess the validity and accuracy of the predicted results by the ANN method, three error functions were used, including the determination coefficient (R^2^), mean absolute error (MAE), and root mean square error (RMSE).

## 3. Results

### 3.1. Numerical Results

The Tesla valve functions as a one-way valve and creates a high-pressure drop in one direction compared to the other direction, making the flow of movement easier in one direction. Therefore, the ratio of Δ*P* in one direction to the other, known as diodicity, is one of the most important parameters in this device. Tesla valves can also be used in thermal applications. In this work, the thermal performance of the device was investigated in addition to its hydraulic performance. Due to the stability of fluid properties and mass flow rate in both flow directions, the ratio of directions temperature difference (TDR) also indicates the heat transfer ratio. The information related to the numerical tests is reported in Table 7, and preliminary analyses can be performed using these data. Based on the results, the maximum values of TDR and PDR are observed in test numbers 8 and 9, respectively. The highest Δ*P* in the reverse direction occurred in test number 7, and the highest Δ*P* in the forward movement occurred in numerical experiment 11. At the same time, the largest difference between the inlet and outlet temperatures in both reverse and forward flow directions was reported in numerical experiment 3.

**Figure 6:**
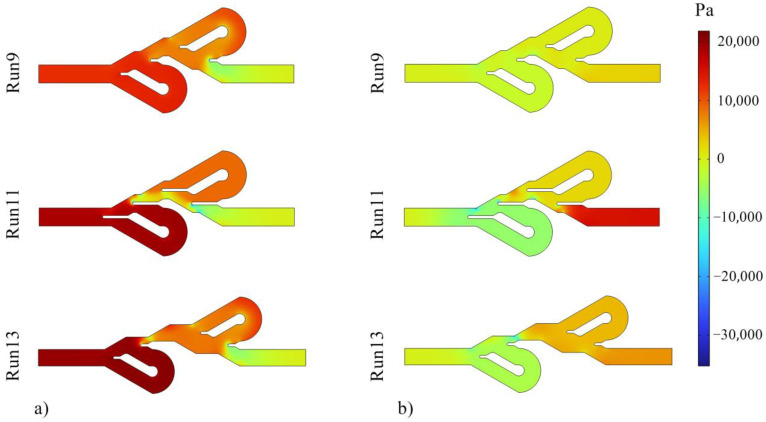
Pressure contours for (**a**) Tesla valves with fluid flow in the reverse direction and (**b**) Tesla valves with fluid flow in the forward direction.

**Figure 7:**
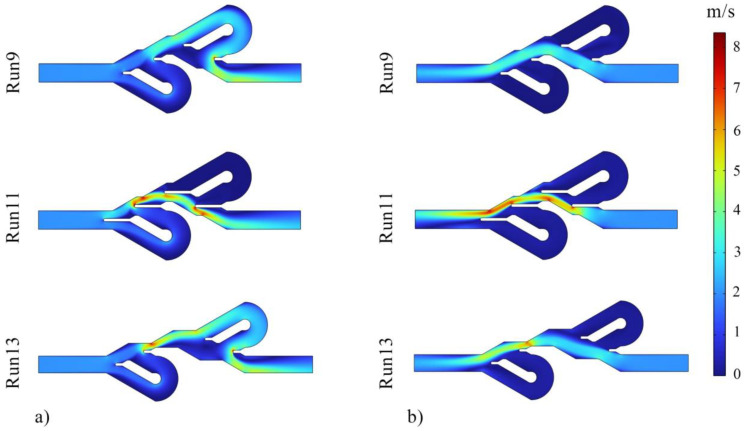
Velocity contours for (**a**) Tesla valves with fluid flow in the reverse direction and (**b**) Tesla valves with fluid flow in the forward direction.

**Figure 8:**
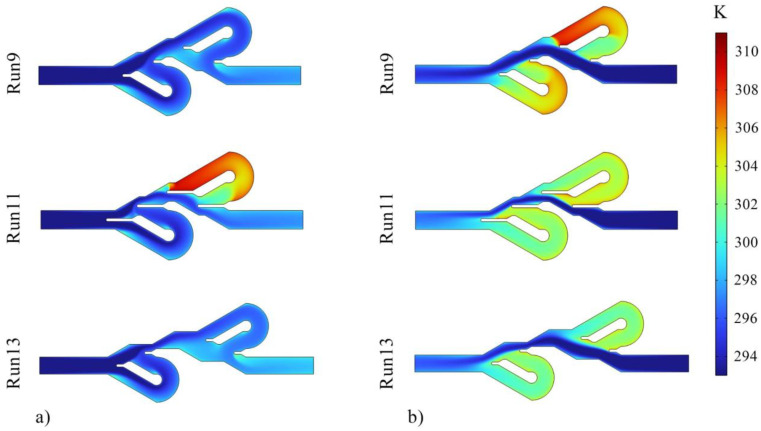
Temperature contours for (**a**) Tesla valves with fluid flow in the reverse direction and (**b**) Tesla valves with fluid flow in the forward direction.

Figure 6 shows the pressure contours, and it can be seen that the inlet pressure of reverse flows is higher than the forward flows direction. Additionally, the fluid pressure in the reverse flow is high due to the longer fluid movement path, more friction, more vortices, and more fluid collisions. Figure 7 displays the velocity contours, and it is evident that in the reverse direction of fluid flow in the Tesla valve, the fluid passes through the bent channels with a higher mass flow rate and velocity than the forward flow. Using these two contours, it was concluded that much flow passes through the bent paths in the reverse direction movement. Therefore, the thermal performance of the device is expected to be higher in the direction of the reverse flow, which is confirmed by Table 6. Figure 8 presents the temperature contours for checking the thermal performance. As shown in this figure, in the Tesla valve with the forward flow, the heat transfer is weaker since less fluid enters the bent channels, and the temperature of the liquid in the bent channels is higher. Of course, in the reverse flow temperature contour of experiment 11, the fluid temperature in the second bent channel is also high due to the geometry of the valve, which causes not much fluid to enter the bend, and the fluid temperature in that section increases. The temperature distribution is generally better in the Tesla valve with reverse flow. The results of tests 9, 11, and 13 are presented in all contours. Experiments 9 and 11 were compared to observe the effect of changing variable *L* on Tesla valve performance, and experiments 9 and 13 were compared to observe the impact of changing variable *S* on valve performance. In the following, the ANN method and predictions of this method were used for a more detailed investigation of the effect of variables and optimization of geometry and responses.

### 3.2. The ANN Results

**Figure 9:**
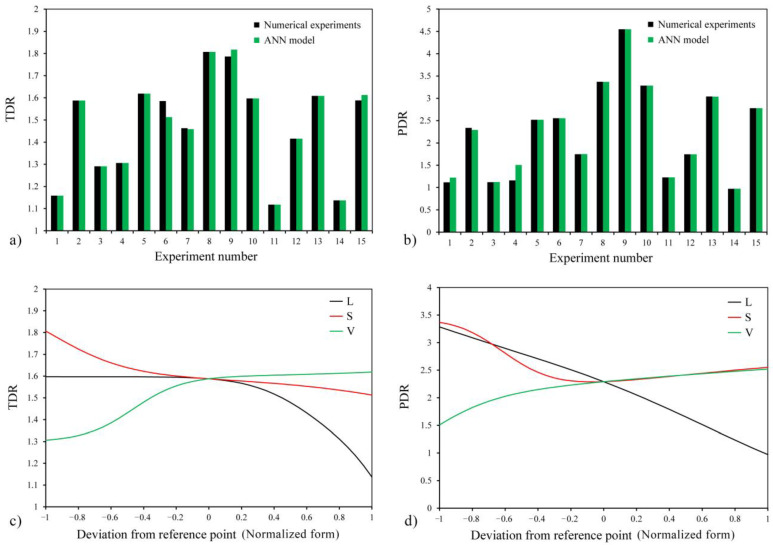
Comparing the results obtained by ANN and numerical methods for (**a**) TDR and (**b**) PDR. Perturbation plots of (**c**) TDR and (**d**) PDR.

In this section, the results of the ANN method are discussed. The predicted results for TDR and PDR responses using the models obtained from the ANN method are compared with the numerical analysis results and presented in Figure 9a,b. It can be seen that the results obtained by both methods are similar. The models for the TDR and PDR responses developed through the ANN method are described in Equations (13) and (15), respectively. The optimized weights and biases of the models related to the responses were obtained using the ANN method and LM algorithm. The R^2^ parameter was examined to assess the accuracy of the models (Table 8), and it was observed that the TDR model correctly predicts 99.1% of the responses. Also, the R^2^ error function with a value of 0.992 for the PDR model indicates that this model can correctly predict 99.2% of responses and has only 0.8% error.

To show the effect of each input variable (*L*, *S*, and *V*) on the output responses, the plots in Figure 9c,d were presented. In these plots, one variable was changed from −1 to +1 levels while the other two remained constant at zero levels. Also, the reference point in these graphs is the point where all three variables are at zero level. As seen in Figure 9c, changes in the *L* variable up to a certain level do not affect TDR, and from that level onwards, it causes TDR to decrease. Changes in the *S* parameter initially cause a decrease in TDR, and the intensity of this reduction gradually decreases. With an increase in the value of *V*, TDR also increases, but the slope of this plot is higher in the initial part, indicating a greater intensity. In the following, the effects of the independent input variables on the response of the diodicity are discussed, shown in Figure 9d. According to the high slope of the *L* parameter graph, it seems that the effect of this variable on the diodicity is more significant than the rest of the variables. After the *L* parameter, the Tesla valve input velocity variable has the most impact on PDR, and this response rises with the increase in velocity.

(13) $$TDR=\left(\left[1.359\;\;\;\;\;0.433\;\;\;\;\;-0.634\;\;\;\;\;-0.073\right]\times \left[\begin{array}{c} \frac{2}{1+exp(-2A_{1})}-1\\ \frac{2}{1+exp(-2A_{2})}-1\\ \frac{2}{1+exp(-2A_{3})}-1\\ \frac{2}{1+exp(-2A_{4})}-1 \end{array}\right]+\left[-0.703\right]+1\right)\times \frac{1.807-1.118}{2}+1.118$$

(14) $$\left[\begin{array}{c} A_{1}\\ A_{2}\\ A_{3}\\ A_{4} \end{array}\right]=\left[\begin{array}{rrr} -2.913 & 1.239 & -0.825\\ -2.622 & -1.047 & 3.314\\ -0.775 & 2.054 & -0.647\\ 1.159 & 0.256 & -1.438 \end{array}\right]\times \left[\begin{array}{c} 2\times \frac{L-2}{8-2}-1\\ 2\times \frac{S-2}{8-2}-1\\ 2\times \frac{V-0.2}{2-0.2}-1 \end{array}\right]+\left[\begin{array}{r} 3.496\\ 1.541\\ 2.027\\ 2.786 \end{array}\right]$$

(15) $$PDR=\left(\left[0.074\;\;\;\;\;-0.981\;\;\;\;\;0.445\;\;\;\;\;-0.472\right]\times \left[\begin{array}{c} \frac{2}{1+exp(-2B_{1})}-1\\ \frac{2}{1+exp(-2B_{2})}-1\\ \frac{2}{1+exp(-2B_{3})}-1\\ \frac{2}{1+exp(-2B_{4})}-1 \end{array}\right]+\left[-0.674\right]+1\right)\times \frac{4.545-0.970}{2}+0.970$$

(16) $$\left[\begin{array}{c} B_{1}\\ B_{2}\\ B_{3}\\ B_{4} \end{array}\right]=\left[\begin{array}{rrr} 1.356 & -0.686 & -1.462\\ 0.807 & -0.265 & -0.205\\ 1.391 & 0.550 & 2.249\\ 1.421 & 2.936 & 0.309 \end{array}\right]\times \left[\begin{array}{c} 2\times \frac{L-2}{8-2}-1\\ 2\times \frac{S-2}{8-2}-1\\ 2\times \frac{V-0.2}{2-0.2}-1 \end{array}\right]+\left[\begin{array}{r} -2.375\\ -0.555\\ 2.542\\ 1.825 \end{array}\right]$$

**Figure 10:**
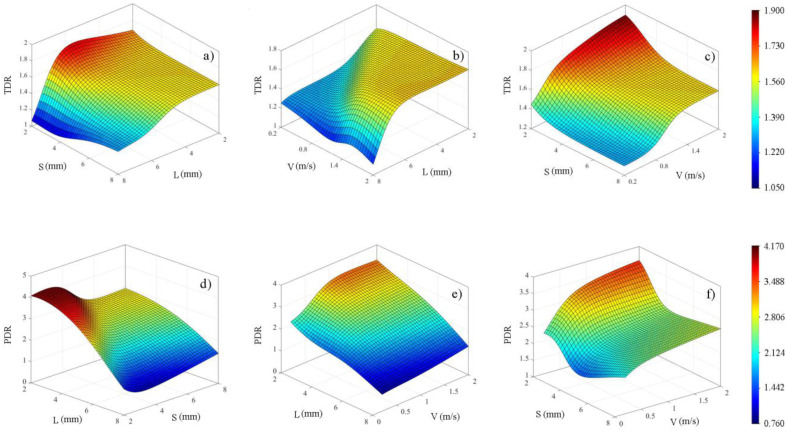
The relationship between (**a**) TDR, *L*, and *S*, (**b**) TDR, *L*, and *V*, (**c**) TDR, *S*, and *V*, (**d**) PDR, *L*, and *S*, (**e**) PDR, *L*, and *V*, (**f**) PDR, *S*, and *V* was illustrated in these 3D plots.

In the following, the two-by-two effects of the variables on the responses were investigated according to Figure 10. Figure 10a shows that as *L* increases, the TDR value remains somewhat stable and then decreases, and the effect of this decrease is more significant at lower *S*. Figure 10b confirms this observation and shows that the effect of the *L* parameter on TDR is more pronounced at higher inlet velocities. Figure 10c reveals that the heat transfer capability of the Tesla valve increases with the increase of the inlet velocity (*V*), and this increase is more significant at lower *S* values. Furthermore, it can be seen that increasing *S* leads to a decrease in TDR. Turning to the three-dimensional plots for PDR response, Figure 10d shows that increasing *L* reduces PDR, and this effect is more pronounced at smaller *S* values. Figure 10e,f demonstrate that increasing the inlet velocity increases PDR, which is more prominent at smaller *L* and *S* values. It is worth mentioning that increasing the *L* parameter leads to a decrease in the diodicity of the Tesla valve.

**Figure 11:**
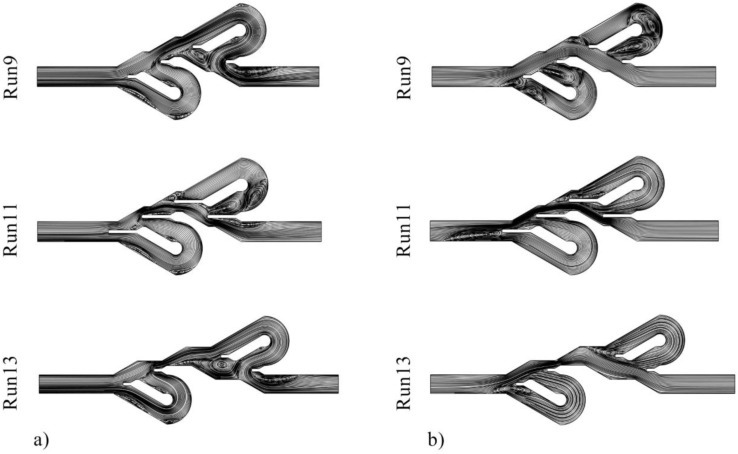
Streamlines of (**a**) reverse flow direction and (**b**) forward flow direction for different numerical experiments.

This section discusses the influence of variables on the performance of the Tesla valve and the analysis of these effects using streamlines (Figure 11). In this figure, the primary issue that can be noticed is that in the direction of forward movement, compression of the lines in the main channel is greater than bent channels, indicating a large fluid flow through this path. While in the reverse flow direction, the fluid flow is spread in all the channels of the Tesla valve. The effect of changing the divider baffle length on PDR can be observed based on the fluid flow streamlines of the 9th and 11th numerical experiments. According to the streamlines in the reverse direction, it is apparent that increasing the divider baffle length causes most of the fluid flow to pass without entering the second bent channel, and this function reduces the Δ*P* and diodicity. However, it should be noted that as *L* increases, the dimensions of the central path reduce, leading to increased Δ*P* in both directions of fluid movement. Comparing the streamlines of experiments 9th and 13th reveals the effect of changing the parameter *S* on the Tesla valve’s performance. It is clearly seen that in the 9th test, more flow enters the bent channels in reverse flow than in the 13th test, and due to this matter, the Δ*P* ratio of reverse flow to the forward flow is higher in this design. Additionally, it should be mentioned that increasing the *S* variable leads to a larger Tesla valve, causing more friction and Δ*P* in both directions.

As mentioned in the previous sections, one of the most important goals of the current research is to provide optimal designs for the Tesla valve in different conditions. The goal was to maximize the values of PDR and TDR, and genetic algorithm and ANN models were used to predict the optimal design parameters. According to Table 9, the designs with maximum PDR and TDR values were predicted, and the predicted results for the values of the responses were also presented. Next, the given Tesla valves were designed and then numerically tested to ensure that the predictions were correct and that these designs had the most optimal response values. The data relating to these numerical analyzes were also reported in Table 9. As can be seen, the predicted results have good accuracy, and at the same time, these designs have the maximum value of diodicity and TDR in reality. The maximum value of TDR in the Tesla valve with the design of *L* = 4.502 mm, *S* = 2 mm, and *V* = 2 m/s was obtained and had a predicted value of 1.908. The numerical results reported the TDR value for this design as 1.862, and it can be said that the ANN model has a 2.5% error in predicting this number. Using this number, it can be argued that in this design, the heat transfer in the reverse direction is 86.2% more than in the forward movement. One of the essential parameters in Tesla valves is diodicity, and it was predicted that the maximum value of this response is achieved in the design of *L* = 2 mm, *S* = 2.048 mm, and *V* = 2 m/s. The predicted and calculated values for PDR were 4.546 and 4.581, respectively, with a low prediction error of 0.8%. The number of 4.581, obtained numerically for diodicity, indicates that the Δ*P* obtained in the reverse direction is 358.1% higher than the Δ*P* obtained in the forward direction. Overall, the approach of using a genetic algorithm and ANN models to predict the optimal design parameters for the Tesla valve proved successful, and the numerical analysis confirmed the accuracy of the predictions.

Using the models provided by ANN, it is possible to predict designs with the best performance at different velocities. In this case, the desired velocity value is entered into the prediction model, and then the genetic algorithm is used to optimize the models. The optimal designs at different inlet velocities for TDR and PDR responses were presented in Table 10 and Table 11, respectively. As observed in the tables, the predicted results agree with those obtained from numerical methods in all cases. Furthermore, upon careful examination of these results, it becomes evident that both responses can be enhanced by increasing the inlet velocity and selecting an appropriate geometric design for the Tesla valve. Next, the performance of the two-stage Tesla valve designed by Bao and Wang [10] was reported in Table 12 to compare with the results of the designs presented in this study. It is evident from the tables that the optimal designs exhibit significantly superior performance compared to the reference design, and this improvement is more visible in PDR.

## 4. Conclusions

This research aimed to optimize the design of a two-stage Tesla valve and study its fluid flow and heat transfer characteristics numerically. The input variables were selected as *L*, *S*, and *V*, and two responses, TDR and PDR, were used to evaluate thermal and hydraulic performances. Then, an ANN was trained for each response using data obtained from numerical experiments to predict the responses for different designs. By using these models, without doing experimental and numerical work and complex calculations, and in the shortest time, the responses related to the design of the desired Tesla valve can be predicted. In the following, the optimal designs of this device for different conditions were presented using models trained by the ANN method and genetic algorithm. According to the findings, the following was determined:

- It was shown that the models obtained using the ANN method could correctly predict the results for the thermal and hydraulic diodicities of the Tesla valve with a determination coefficient of 99.1% and 99.2%, respectively;
- It was found that increasing the length of the divider baffle decreases PDR. Also, increasing this variable from a specific limit reduces the value of the TDR response;
- The fluid inlet velocity parameter positively affects the responses, and generally, with its increase, PDR and TDR also increase;
- The highest value of diodicity was predicted for the Tesla valve with *L* = 2 mm, *S* = 2.048 mm, and *V* = 2 m/s parameters. This prediction was confirmed by performing numerical tests. The predicted diodicity value for this design is 4.546, and numerical tests reported this number to be 4.581. The prediction error of this response is very low and equal to 0.8%. This Tesla valve with a PDR of 4.581 indicates that the pressure drop in the reverse flow direction is 358.1% more than in the forward flow direction;
- The most optimal TDR response value was predicted to be 1.908, obtained in the condition of *L* = 4.502 mm, *S* = 2 mm, and *V* = 2 m/s. Numerical tests were performed on the designed Tesla valve, and the actual TDR was 1.862, a 2.5% difference from the predicted value. TDR with this value shows that the Tesla valve with the mentioned design has 86.2% more heat transfer in the reverse direction.

## Figures and Tables

**Table 1:** The thermophysical characteristics of the water at 293.15 K [10].

Properties	Value
$\rho \;(\mathrm{kg}/m^{3}$)	998.2
$\mu \;\left(\mathrm{Pa}\cdot s\right)$	1.003 × 10^−3^
$C_{p}\;\left(J/\mathrm{kg}\cdot K\right)$	4182
$\lambda \;\left(W/m\cdot K\right)$	0.6

**Table 2:** Constant parameters related to standard *k-ε* turbulence model.

Constant Parameter	*σ_k_*	*σ_ε_*	*C_ε1_*	*C_ε2_*	*C_μ_*
Value	1	1.30	1.44	1.92	0.09

**Table 3:** Mesh independence validation for tesla valve with six-stage and reverse flow at conditions *V* = 0.2 m/s, *T_in_* = 274.15 K, and *T_w_* = 368.15 K.

Number of Elements per 1 mm^2^	Solve Time	Δ*T_r_*		Δ*P_r_*	
		Value (K)	Difference (%)	Value (Pa)	Difference (%)
3.89	00:00:48	24.210	21.45	1905	7.15
22.68	00:03:38	29.320	4.87	1835	0.83
50.44	00:25:13	30.440	1.23	1828	0.44
88.58	00:39:27	30.820	-	1820	-

**Table 4:** The value of *y+* in different turbulence models at *Z* = 300 mm, *T_in_* = 274.15 K, and *T_w_* = 368.15 K conditions.

Turbulence Model	Wall Model	Value of *y+*		Suitability Situation
		Reverse Flow	Forward Flow	
Standard *k-ε*	Wall functions	41.910	40.749	suitable
Realizable *k-ε*	Wall functions	41.784	45.745	suitable
*SST k-ω*	LRN	29.211	36.539	unsuitable
*k-ω*	LRN	39.675	42.715	unsuitable

**Table 5:** Selected variables and levels.

Variable	Variable Levels		
	−1	0	+1
L (mm)	2	5	8
S (mm)	2	5	8
V (m/s)	0.2	1.1	2

**Table 6:** Selected numerical experiments to perform numerical simulations.

Numerical Experiment Number	Position Used in Machine Learning	Variables		
		*L* (mm)	*S* (mm)	*V* (m/s)
1	Train	8	2	0.2
2	Train	5	5	1.1
3	Train	8	8	0.2
4	Validation	5	5	0.2
5	Train	5	5	2
6	Train	5	8	1.1
7	Train	8	8	2
8	Train	5	2	1.1
9	Train	2	2	2
10	Train	2	5	1.1
11	Test	8	2	2
12	Train	2	8	0.2
13	Train	2	8	2
14	Validation	8	5	1.1
15	Test	2	2	0.2

**Table 7:** Data and results related to numerical method experiments.

Numerical Experiment Number	Numerical Simulation Data					
	Δ*T_f_* (K)	Δ*T_r_* (K)	Δ*P_f_* (Pa)	Δ*P_r_* (Pa)	TDR	PDR
1	6.31	7.31	254.28	284.32	1.158	1.118
2	3.83	6.08	2479.4	5790.9	1.587	2.336
3	6.94	8.96	240.18	269.04	1.291	1.120
4	5.46	7.13	128.58	149.04	1.306	1.159
5	3.46	5.6	7259.5	18,286	1.618	2.519
6	4.22	6.69	2995.2	7646.8	1.585	2.553
7	4.41	6.45	14,233	24,887	1.463	1.749
8	3.47	6.27	2260.1	7614.9	1.807	3.369
9	2.67	4.77	2767.3	12,578	1.787	4.545
10	3.50	5.59	1407.2	4622.9	1.597	3.285
11	4.16	4.65	15,159	18,580	1.118	1.226
12	5.75	8.14	120.32	209.9	1.416	1.745
13	3.55	5.71	6522.3	19,819	1.608	3.039
14	4.45	5.06	4414.8	4283.5	1.137	0.970
15	4.49	7.13	48.879	135.79	1.588	2.778

**Table 8:** Nonlinear error functions for prediction models obtained by ANN method.

Error Function	Mathematical Form	ANN	
		TDR	PDR
R^2^	$1-\frac{\sum_{i=1}^{m}\left(r_{num}-r_{pred}\right)}{\sum_{i=1}^{m}{\left(r_{num}-\bar{r_{pred}}\right)}^{2}}$	0.991	0.992
MAE	$\frac{1}{m}\sum_{i=1}^{m}\left|r_{pred}-r_{num}\right|$	0.009	0.034
RMSE	$\sqrt{\frac{1}{m}\sum_{i=1}^{m}{\left(r_{pred}-r_{num}\right)}^{2}}$	0.021	0.095

**Table 9:** Comparison of predicted and numerical data for the predicted optimal design of the Tesla valve.

Optimized Parameter	Optimal Design			ANN Predicted Data		Numerical Data	
	L (mm)	S (mm)	V (m/s)	TDR	PDR	TDR	PDR
TDR (maximization)	4.502	2	2	1.908	3.848	1.862	3.835
PDR (maximization)	2	2.048	2	1.810	4.546	1.776	4.581

**Table 10:** Optimal geometric design for TDR optimization at different velocities.

Inlet Velocity (m/s)	Optimal Geometric Design		ANN Predicted Data		Numerical Data	
	L (mm)	S (mm)	TDR	PDR	TDR	PDR
0.2	2.066	2	1.613	2.766	1.594	2.762
0.5	4.004	2	1.663	2.963	1.740	3.014
1	5.504	2	1.801	3.040	1.794	3.092
1.5	5.117	2	1.860	3.415	1.838	3.448
2	4.502	2	1.908	3.848	1.862	3.835

**Table 11:** Optimal geometric design for PDR optimization at different velocities.

Inlet Velocity (m/s)	Optimal Geometric Design		ANN Predicted Data		Numerical Data	
	L (mm)	S (mm)	TDR	PDR	TDR	PDR
0.2	2	3.107	1.586	2.837	1.664	2.787
0.5	2	3.284	1.599	3.108	1.678	3.360
1	2.270	2.978	1.629	4.034	1.745	3.976
1.5	2	2.453	1.691	4.449	1.768	4.398
2	2	2.048	1.810	4.546	1.776	4.581

**Table 12:** Performance of the two-stage Tesla valve presented in Bao and Wang’s work at different velocities.

Inlet Velocity (m/s)	Designed by Bao and Wang [10]	
	TDR	PDR
0.2	1.323	1.310
0.5	1.354	1.611
1	1.358	1.787
1.5	1.375	1.845
2	1.386	1.874

## Data Availability

The data presented in this study are available on logical request from the corresponding author.

## Abbreviations

Nomenclature	
*b*	bias
*C_p_*	specific heat capacity (J/kg·K)
*k*	turbulent kinetic energy
*L*	divider baffle length (mm)
*Ma*	Mach number
*m*	number of tests
$\dot{m}$	mass flow rate (g/min)
Δ*P*	pressure drop (Pa)
*Pr*	Prandtl number
*r*	response
*S*	step length (mm)
*T*	temperature (K)
*V*	velocity (m/s)
*V_τ_*	friction velocity (m/s)
*W*	weight
*Z*	height of fluid column (mm)
Symbols	
*ρ*	density (kg/m^3^)
*µ*	dynamic viscosity (Pa·s)
*λ*	thermal conductivity (W/m·K)
*ε*	energy dissipation rate
Subscripts	
*exp*	experimental data
*f*	forward flow direction
*in*	inlet
*num*	numerical data
*out*	outlet
*pred*	predicted data
*r*	reverse flow direction
*t*	turbulence
*w*	wall

## References

[1] Porwal P.R., Thompson S.M., Walters D.K., Jamal T. (2018). Heat Transfer and Fluid Flow Characteristics in Multistaged Tesla Valves. Numer. Heat Transf. Part A Appl..

[2] Liu Z., Shao W.Q., Sun Y., Sun B.H. (2022). Scaling Law of the One-Direction Flow Characteristics of Symmetric Tesla Valve. Eng. Appl. Comput. Fluid Mech..

[3] Doddamani H., Samad A. (2023). Dynamic Performance of a Fluidic Diode Subjected to Periodic Flow. Ocean Eng..

[4] Hithaish D., Siddique M.H., Samad A. (2022). A Pareto Optimal Front of Fluidic Diode for a Wave Energy Harnessing Device. Ocean Eng..

[5] Qian J.Y., Chen M.R., Liu X.L., Jin Z.J. (2019). A Numerical Investigation of the Flow of Nanofluids through a Micro Tesla Valve. J. Zhejiang Univ. Sci. A.

[6] Nigro A., Algieri A., De Bartolo C., Bova S. (2017). Fluid Dynamic Investigation of Innovative Intake Strategies for Multivalve Internal Combustion Engines. Int. J. Mech. Sci..

[7] Yang X., Liu Z., Liu Z., Feng Z., Simon T. (2019). Turbine Platform Phantom Cooling from Airfoil Film Coolant, with Purge Flow. Int. J. Heat Mass Transf..

[8] Nowak A.J. (2014). Selected papers presented during the Numerical Heat Transfer 2012 International Conference (NHT2012) held on 4–6 September 2012 in Wroclaw, Poland. Int. J. Numer. Methods Heat Fluid Flow.

[9] Pakatchian M.R., Saeidi H., Ziamolki A. (2020). CFD-Based Blade Shape Optimization of MGT-70(3)Axial Flow Compressor. Int. J. Numer. Methods Heat Fluid Flow.

[10] Bao Y., Wang H. (2022). Numerical Study on Flow and Heat Transfer Characteristics of a Novel Tesla Valve with Improved Evaluation Method. Int. J. Heat Mass Transf..

[11] Liosis C., Sofiadis G., Karvelas E., Karakasidis T., Sarris I. (2022). A Tesla Valve as a Micromixer for Fe3O4 Nanoparticles. Processes.

[12] Jin Z.J., Gao Z.X., Chen M.R., Qian J.Y. (2018). Parametric Study on Tesla Valve with Reverse Flow for Hydrogen Decompression. Int. J. Hydrogen Energy.

[13] Qian J.Y., Wu J.Y., Gao Z.X., Wu A., Jin Z.J. (2019). Hydrogen Decompression Analysis by Multi-Stage Tesla Valves for Hydrogen Fuel Cell. Int. J. Hydrogen Energy.

[14] Lu Y., Wang J., Liu F., Liu Y., Wang F., Yang N., Lu D., Jia Y. (2022). Performance Optimisation of Tesla Valve-Type Channel for Cooling Lithium-Ion Batteries. Appl. Therm. Eng..

[15] Monika K., Chakraborty C., Roy S., Sujith R., Datta S.P. (2021). A Numerical Analysis on Multi-Stage Tesla Valve Based Cold Plate for Cooling of Pouch Type Li-Ion Batteries. Int. J. Heat Mass Transf..

[16] de Vries S.F., Florea D., Homburg F.G.A., Frijns A.J.H. (2017). Design and Operation of a Tesla-Type Valve for Pulsating Heat Pipes. Int. J. Heat Mass Transf..

[17] Qian J.Y., Chen M.R., Gao Z.X., Jin Z.J. (2019). Number and Energy Loss Analysis inside Multi-Stage Tesla Valves for Hydrogen Decompression. Energy.

[18] Yang A.S., Chuang F.C., Chen C.K., Lee M.H., Chen S.W., Su T.L., Yang Y.C. (2015). A High-Performance Micromixer Using Three-Dimensional Tesla Structures for Bio-Applications. Chem. Eng. J..

[19] Sun L., Li J., Xu H., Ma J., Peng H. (2022). Numerical Study on Heat Transfer and Flow Characteristics of Novel Microchannel Heat Sinks. Int. J. Therm. Sci..

[20] Esen H., Inalli M., Sengur A., Esen M. (2008). Performance Prediction of a Ground-Coupled Heat Pump System Using Artificial Neural Networks. Expert Syst. Appl..

[21] Sivaprasad H., Lekkala M.R., Latheef M., Seo J., Yoo K., Jin C., Kim D.K. (2023). Fatigue Damage Prediction of Top Tensioned Riser Subjected to Vortex-Induced Vibrations Using Artificial Neural Networks. Ocean Eng..

[22] Ahmad I., M’zoughi F., Aboutalebi P., Garrido I., Garrido A.J. (2023). Fuzzy Logic Control of an Artificial Neural Network-Based Floating Offshore Wind Turbine Model Integrated with Four Oscillating Water Columns. Ocean Eng..

[23] Olabi A.G., Abdelkareem M.A., Semeraro C., Al Radi M., Rezk H., Muhaisen O., Al-Isawi O.A., Sayed E.T. (2023). Artificial Neural Networks Applications in Partially Shaded PV Systems. Therm. Sci. Eng. Prog..

[24] Yulia F., Chairina I., Zulys A., Nasruddin (2021). Multi-Objective Genetic Algorithm Optimization with an Artificial Neural Network for CO2/CH4 Adsorption Prediction in Metal–Organic Framework. Therm. Sci. Eng. Prog..

[25] Gao J., Hu Z., Yang Q., Liang X., Wu H. (2022). Fluid Flow and Heat Transfer in Microchannel Heat Sinks: Modelling Review and Recent Progress. Therm. Sci. Eng. Prog..

[26] Mollalo A., Rivera K.M., Vahedi B. (2020). Artificial Neural Network Modeling of Novel Coronavirus (COVID-19) Incidence Rates across the Continental United States. Int. J. Environ. Res. Public Health.

[27] Abiodun O.I., Kiru M.U., Jantan A., Omolara A.E., Dada K.V., Umar A.M., Linus O.U., Arshad H., Kazaure A.A., Gana U. (2019). Comprehensive Review of Artificial Neural Network Applications to Pattern Recognition. IEEE Access.

[28] Polat M.E., Cadirci S. (2022). Artificial Neural Network Model and Multi-Objective Optimization of Microchannel Heat Sinks with Diamond-Shaped Pin Fins. Int. J. Heat Mass Transf..

[29] Kanesan J., Arunasalam P., Seetharamu K.N., Azid I.A. Artificial Neural Network Trained, Genetic Algorithms Optimized Thermal Energy Storage Heatsinks for Electronics Cooling. Proceedings of the ASME 2005 Pacific Rim Technical Conference and Exhibition on Integration and Packaging of MEMS, NEMS, and Electronic Systems collocated with the ASME 2005 Heat Transfer Summer Conference.

[30] Mahmoudabadbozchelou M., Eghtesad A., Jamali S., Afshin H. (2020). Entropy Analysis and Thermal Optimization of Nanofluid Impinging Jet Using Artificial Neural Network and Genetic Algorithm. Int. Commun. Heat Mass Transf..

[31] Kuang Y., Han F., Sun L., Zhuan R., Wang W. (2021). Saturated Hydrogen Nucleate Flow Boiling Heat Transfer Coefficients Study Based on Artificial Neural Network. Int. J. Heat Mass Transf..

[32] Heng S.Y., Asako Y., Suwa T., Nagasaka K. (2019). Transient Thermal Prediction Methodology for Parabolic Trough Solar Collector Tube Using Artificial Neural Network. Renew. Energy.

[33] Ermis K., Erek A., Dincer I. (2007). Heat Transfer Analysis of Phase Change Process in a Finned-Tube Thermal Energy Storage System Using Artificial Neural Network. Int. J. Heat Mass Transf..

[34] Xie G., Sunden B., Wang Q., Tang L. (2009). Performance Predictions of Laminar and Turbulent Heat Transfer and Fluid Flow of Heat Exchangers Having Large Tube-Diameter and Large Tube-Row by Artificial Neural Networks. Int. J. Heat Mass Transf..

[35] Beigmoradi S., Hajabdollahi H., Ramezani A. (2014). Multi-Objective Aero Acoustic Optimization of Rear End in a Simplified Car Model by Using Hybrid Robust Parameter Design, Artificial Neural Networks and Genetic Algorithm Methods. Comput. Fluids.

[36] Li B., Lee Y., Yao W., Lu Y., Fan X. (2020). Development and Application of ANN Model for Property Prediction of Supercritical Kerosene. Comput. Fluids.

[37] George A., Poguluri S.K., Kim J., Cho I.H. (2022). Design Optimization of a Multi-Layer Porous Wave Absorber Using an Artificial Neural Network Model. Ocean Eng..

[38] Zhu K., Shi H., Han M., Cao F. (2022). Layout Study of Wave Energy Converter Arrays by an Artificial Neural Network and Adaptive Genetic Algorithm. Ocean Eng..

[39] Vaferi K., Vajdi M., Nekahi S., Nekahi S., Sadegh Moghanlou F., Azizi S., Shahedi Asl M. (2023). Numerical Simulation of Cooling Performance in Microchannel Heat Sinks Made of AlN Ceramics. Microsyst. Technol..

[40] Li S., Li C., Li Z., Xu X., Ye C., Zhang W. (2020). Design Optimization and Experimental Performance Test of Dynamic Flow Balance Valve. Eng. Appl. Comput. Fluid Mech..

[41] Kuzmin D., Mierka O., Turek S. (2007). On the Implementation of the Fr-Fturbulence Model in Incompressible Flow Solvers Based on a Finite Element Discretisation. Int. J. Comput. Sci. Math..

[42] Savicki D.L., Goulart A., Becker G.Z. (2021). A Simplified K- ϵ Turbulence Model. J. Brazilian Soc. Mech. Sci. Eng..

[43] Micale D., Ferroni C., Uglietti R., Bracconi M., Maestri M. (2022). Computational Fluid Dynamics of Reacting Flows at Surfaces: Methodologies and Applications. Chemie Ing. Tech..

[44] Demidov D., Mu L., Wang B. (2021). Accelerating Linear Solvers for Stokes Problems with C++ Metaprogramming. J. Comput. Sci..

[45] Schenk O., Gärtner K., Fichtner W., Stricker A. (2001). PARDISO: A High-Performance Serial and Parallel Sparse Linear Solver in Semiconductor Device Simulation. Futur. Gener. Comput. Syst..

[46] Vaferi K., Vajdi M., Nekahi S., Nekahi S., Sadegh Moghanlou F., Shahedi Asl M., Mohammadi M. (2021). Thermo-Mechanical Simulation of Ultrahigh Temperature Ceramic Composites as Alternative Materials for Gas Turbine Stator Blades. Ceram. Int..

[47] Salim S.M., Ariff M., Cheah S.C. (2010). Wall Y+ Approach for Dealing with Turbulent Flows over a Wall Mounted Cube. Prog. Comput. Fluid Dyn. Int. J..

[48] Blocken B., Stathopoulos T., Carmeliet J. (2007). CFD Simulation of the Atmospheric Boundary Layer: Wall Function Problems. Atmos. Environ..

[49] Klewicki J., Saric W., Marusic I., Eaton J. (2007). Wall-Bounded Flows. Springer Handbook of Experimental Fluid Mechanics.

[50] Hou D., Chen D., Wang X., Wu D., Ma H., Hu X., Zhang Y., Wang P., Yu R. (2020). RSM-Based Modelling and Optimization of Magnesium Phosphate Cement-Based Rapid-Repair Materials. Constr. Build. Mater..

